# An Evaluation Index of Fracability for Reservoir Rocks Based on Fracture Process Zone

**DOI:** 10.3390/ma15238485

**Published:** 2022-11-28

**Authors:** Hongran Chen, Jingrui Niu, Mengyang Zhai

**Affiliations:** 1 Key Laboratory of Shale Gas and Geoengineering, Institute of Geology and Geophysics, Chinese Academy of Sciences, Beijing 100029, China; 2 Innovation Academy for Earth Science, CAS, Beijing 100029, China; 3 Chinese Society for Rock Mechanics and Engineering, Beijing 100029, China

**Keywords:** fracability, fracture process zone, crack tolerance, chevron notched disk, discrete element method

## Abstract

A reliable evaluation method for the fracability (i.e., ability to generate abundant cracks) of reservoir rocks is a critical issue for maximum hydraulic fracturing efficiency. Most previous fracability indices lacked enough rationality and practicability, and thus could not consistently provide a reliable evaluation. We suggest a new fracability index called crack tolerance, which is represented by the maximum radius of the fracture process zone at the crack tip of a cracked chevron notched Brazilian disk specimen, which corresponds to the critical state for unstable propagation of the notched crack. In experiments and simulations based on the discrete element method, we showed quantitative methods to conveniently determine the value of the crack tolerance and showed that specimens with a greater crack tolerance generated more cracks before rupture and had complex morphologies, which would indicate stronger fracability. The crack tolerance can well characterize the effects of structural and loading conditions, including the grain size heterogeneity, bedding orientation, and environmental temperature, on fracability, and the inherent heterogeneity of rock is the physical basis for it as a fracability evaluation index. Our studies showed the rationality and practicability of this index and provide hints for how to produce abundant complex cracks in reservoirs.

## 1. Introduction

Human beings’ mining engineering and energy resource exploitation extensively involve the generation and propagation of cracks within rock materials. Hydraulic fracturing is widely used to enhance the fluid conductivity of reservoirs of oil, gas, and geothermal resources. A reliable evaluation of the rock fracability (i.e., ability to generate abundant cracks) is important for hydraulic fracturing [1].

Brittleness, which is generally viewed as a property (or ability) of solid material that ruptures with little appreciable permanent deformation, has long been considered approximately equivalent to fracability, because it shows empirical relevance to the possibility of crack propagation: reservoir comprising brittle rocks usually responds well to stimulation, whereas preexisting and hydraulic fractures tend to heal rather than to propagate in a less brittle reservoir. This is probably attributed to less energy consumed by the ductile deformation of brittle rock materials [2].

In the past decades, a variety of brittleness indices have been developed to evaluate its effect [3,4], which can be classified into several broad categories:

(1) Based on mineral composition (e.g., [5]), especially the weight or volume proportion of hard minerals such as quartz: a positive correlation seems to exist between the brittleness and mineral contents of rocks. However, such indices do not consider many other factors that also contribute to brittleness, such as grain size and loading conditions.
(2) Based on elastic parameters (e.g., [6]): for example, rocks with a large Young’s modulus and small Poisson’s ratio are assumed more brittle. However, such indices can be controversial because many laboratories and in situ observations [7,8] contradict this assumption.
(3) Based on strength: for example, one such index is the ratio of tensile and compressive strengths [9]. Such indices are easily measured, but they lack a physical correlation to brittleness and cracking propagation mechanisms. Thus, these indices may return similar values for various types of rocks with different levels of brittleness.
(4) Based on characteristics of the stress–strain curve such as the relative stress drop, post-peak modulus, and various combinations [10,11]: these indices characterize rock brittleness well and are widely used in predicting the rockburst proneness. However, high brittleness does not consistently represent strong fracability because brittle rock can also act as a barrier to hydraulic fracturing [12].

In summary, many brittleness indices currently popular in fracability evaluation for reservoir lacks mechanical relevance to the rock cracking process. On the other hand, the evaluation indices used in other areas (e.g., those used to estimate rock cuttability [13]) are usually inapplicable for reservoir fracability evaluation owing to the essential differences of physical meaning between brittleness and fracability. Thus far, few evaluation indices of rock fracability meet the following requirements [3]:

(1) Has a firm physical basis;
(2) Consider the heterogeneity of rock material;
(3) Be convenient to measure;
(4) Characterize the effects of loading conditions.

To address this issue, we propose a new evaluation index for rock fracability that we call the *crack tolerance*. See Section 2 for its definition. Section 3 and Section 4 show the experimental measurement of this new index and the corresponding numerical simulation results, respectively, to demonstrate the rationality of the index. Based on these analyses, the effects of several characteristics of the rock materials on the crack tolerance are discussed in Section 5.

This study demonstrated the physical rationality of the crack tolerance as an evaluation index and analyzed the effects of the rock structure and loading conditions on the crack tolerance in an effort to extend our understanding of rock fracability and provide hints for how to produce more cracks in the reservoir.

## 2. Fracture Process Zone and Crack Tolerance

**Figure 1:**
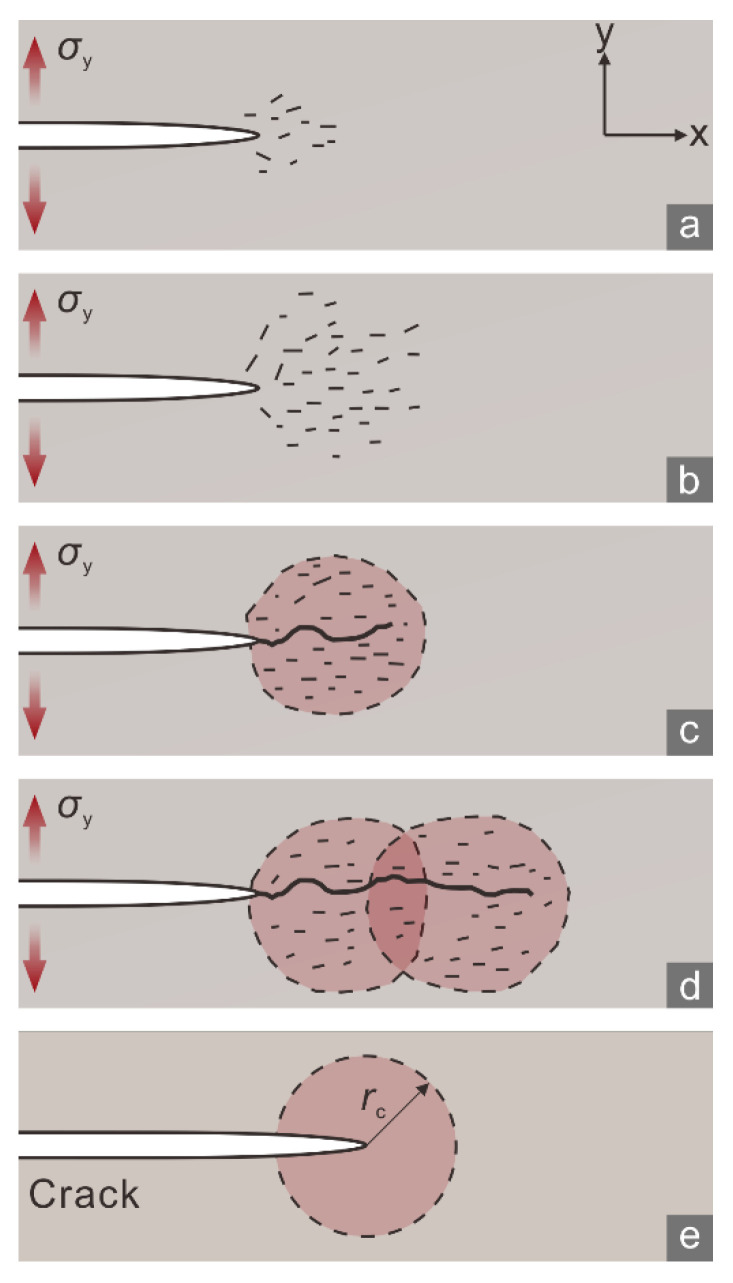
(**a**–**d**) Formation of a fracture process zone (FPZ), shaded in red, and the propagation of macro fractures under the tensile stress *σ*_y_ (denoted by red arrows). (**e**) Schematic of an FPZ with the size *r*_c_.

Numerous researchers have revealed that the propagation of macroscopic cracks within rock under tension is attributed to progressive generation, interaction, and nucleation of micro-cracks from the macroscopic crack tips as follows [14]. When the imposed tensile load is small, only a few independent micro-cracks can arise around each crack tip (Figure 1a). As the tensile load increases, the distribution range of the micro-cracks expands, and their density increases. They interact with each other and coalesce (Figure 1b) to cause a gradual macroscopic propagation of the preexisting crack (Figure 1c,d). These micro-cracks indicate nonlinear deformation in the region around a crack tip preceding crack unstable propagation, which is referred to as a fracture process zone (FPZ) [14].

Crack propagation in tensile mode is most common in hydraulic fracturing because the effect of hydraulic pressure imposed on the crack surface approximates remote tensile stress in nature; additionally, rocks have a much lower tensile strength than compressive and shear strengths. Thus, cracks easily propagate driven by an injected fluid. The principal stresses at a tensile crack tip can be described as [15]

(1) $$\left[\begin{array}{c} \sigma_{1}\\ \sigma_{2} \end{array}\right]=\frac{K_{I}}{\sqrt{2\pi r}}\left[\begin{array}{c} \cos\frac{\theta }{2}\left(1+\sin\frac{\theta }{2}\right)\\ \cos\frac{\theta }{2}\left(1-\sin\frac{\theta }{2}\right) \end{array}\right]$$

where *σ*_1_ and *σ*_2_ are maximum and intermediate principal stresses, *K*_I_ is tensile stress intensity factor, *r* and *θ* are polar radius and polar angle for polar coordinate system from the tip. Note that the minimum principal stress not listed here equals to zero. The range of FPZ (i.e., its size) is calculated based on the hypothesis that nonlinear deformation occurs within a region around crack tip when the local stress state satisfies a certain criterion (e.g., tensile strength criterion for rock materials, von Mises criterion for metal materials). The tensile cracks are assumed to propagate parallel to their own plane (i.e., *θ* = 0) when the *σ*_1_ reaches the tensile strength of the rock (*σ*_t_), because the critical state of crack propagation is attained, which corresponds to the maximum size of the FPZ:

(2) $$\sigma_{t}=\frac{K_{\mathrm{IC}}}{\sqrt{2\pi r_{c}}}\cos0\left(1+\sin0\right)$$

which leads to

*r*_c_ = (*K*_IC_/*σ*_t_)^2^/(2π), (3)

where *K*_IC_ is the tensile fracture toughness, and *r*_c_ is the maximum FPZ size. In this context, the FPZ is represented by a circle centered on a fracture tip [14] (Figure 1e), and *r*_c_ is the radius of a circular FPZ.

A large *r*_c_ would indicate that micro-cracks are distributed within a large FPZ in front of a preexisting crack tip. It would also suggest a considerable number of micro-cracks within the FPZ because a preexisting crack will not propagate until the micro-crack density is high enough to reach a critical level [16]. Therefore, *r*_c_ may characterize the maximum number of micro-cracks generated in the preparation stage for macroscopic crack propagation. In other words, *r*_c_ can be used to indicate the ability of a rock to tolerate micro-cracks before crack unstable propagation. For this reason, we refer to *r*_c_ as the *crack tolerance*. The crack morphology may also depend on the crack tolerance because a large *r*_c_ would indicate an extensive distribution of micro-cracks, which would likely result in irregular and branch cracks.

The concepts of the FPZ and *r*_c_ derive from the propagation process of a single crack with specific boundary conditions. Nevertheless, this process represents the inherent mechanical rule of crack generation within rocks because each crack started as an FPZ. Based on this understanding, the crack tolerance may reflect the potential of a given rock stratum to generate abundant cracks. Recent studies [17,18] have shown that rock specimens with a larger FPZ produce more fragments, which suggests greater fracability and provides evidence supporting our hypothesis. The maximum FPZ radius has exhibited dependence on the structure [19,20] and loading conditions [21] of rock. Thus, we conducted experiments and numerical simulations to analyze their effects on crack tolerance and demonstrate its rationality as an evaluation index.

## 3. Experiments

### 3.1. Specimens

We used marble, shale, and sandstone collected from Xishan, Beijing for experiments because marble was observed in some geothermal reservoirs, and shale and sandstone are representative lithologies comprising oil and gas reservoirs. The marble was divided into types A and J (Table 1): marble A totally constituted by calcite had a greater average grain size and was more heterogeneous as defined by Han et al. [22], and marble J mainly consisting of dolomite had an equigranular texture. The microscopy observation and X-ray diffraction (XRD) analysis showed that the shale with fine grains consisted of quartz (55.4%), plagioclase (6.2%), and clay minerals (38.4%, brown grains in Table 1). The quartz and clay minerals were alternatively layered. The sandstone consisted of quartz (69.5%), plagioclase (22.1%), and potassium feldspar (8.4%), and these xenomorphic grains have similar sizes (~2 mm). Most plagioclase grains experienced sericitization.

### 3.2. Experimental Methodology

The cracked chevron notched Brazilian disk (CCNBD) test involves the formation of FPZs at the two tips of a prefabricated notched crack, which is analogous to a natural crack. Therefore, the CCNBD test is applicable to evaluating crack tolerance. According to Equation (3), quantifying the crack tolerance requires determining the tensile fracture toughness *K*_IC_ and tensile strength *σ*_t_, which are measured by the CCNBD and Brazilian disk (BD) tests, respectively, as recommended by the International Society for Rock Mechanics (ISRM) and American Society of Testing Materials (ASTM).

**Figure 2:**
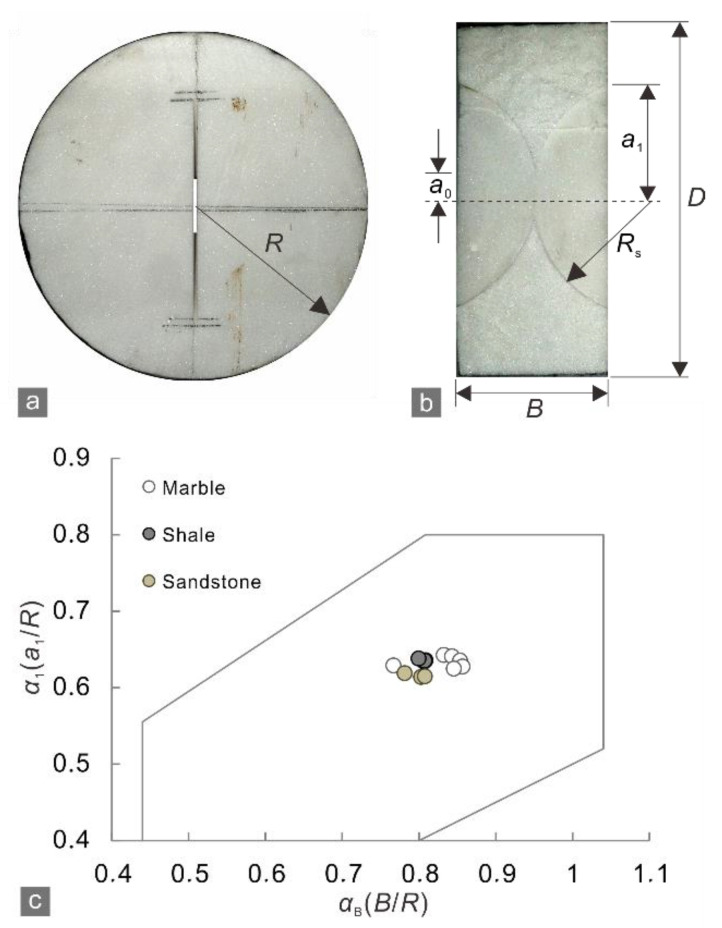
(**a**) Orthographic and (**b**) side profiles of a marble CCNBD specimen; (**c**) valid range for dimensionless parameters *α*_1_ and *α*_B_ (outlined in gray) [23] and the distribution of parameter values for all of the prepared CCNBD specimens. Geometric parameters: diameter *D* = 75 mm, radius *R* = 37.5 mm, thickness *B* = 30 mm, saw radius *R*_s_ = 25 mm, initial chevron notched crack length *a*_0_ = 8.45 mm, and final chevron notched crack length *a*_1_ = 23.5 mm.

The notched crack of each CCNBD specimen was created by a 1 mm thick circular diamond saw. To ensure cutting accuracy, the expected locations of the circular center and the initial and final chevron notched cracks were marked on each disk. We measured the actual values of the parameters shown in Figure 2a,b and confirmed that the dimensionless parameters *α*_1_ and *α*_B_ of all CCNBD specimens were within the valid range (Figure 2c). The method reported by Fowell et al. [23] was used to calculate the *K*_IC_:

*K*_IC_ = *P*_max_Y^*^_min_/*BR*^1/2^, (4)

where *P*_max_ is the peak applied axial load in the CCNBD test and Y^*^_min_ is the critical dimensionless stress intensity value. This is determined by

Y^*^ = *u* · exp (*v* · *α*_1_), (5)

where *u* and *v* are geometric constants that are determined by *α*_0_ and *α*_B_ as reported by Fowell et al. [23].

The thickness (*B*’) and diameter (*D*’) of the BD specimens were set identical to those of the CCNBD specimens to eliminate the size effect on the calculated crack tolerance. The *B*’-to-*D*’ ratio was within the range recommended by the ASTM of 0.2–0.75 [24]. The *σ*_t_ was calculated as follows:

*σ*_t_ = 2*P*’_max_/π*B*’*D*’, (6)

where *P*’_max_ is the peak applied axial load in the BD test.

**Figure 3:**
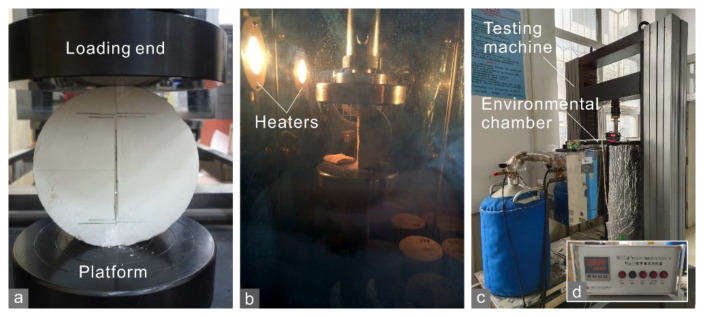
(**a**) CCNBD test on a marble A specimen at room temperature; (**b**) BD test on a sandstone specimen in the chamber at 125 °C; (**c**) MTS servo-control testing machine, SNAS GDS-300 environmental chamber, and (**d**) WK650 temperature controller.

Each CCNBD or BD test (Figure 3a,b) was performed at a constant displacement rate of 0.06 mm/min by an MTS servo-control testing machine (series CMT) with a maximum loading force of 100 kN. This machine is equipped with an SNAS GDS-300 environmental chamber controlled by a WK650 controller (Figure 3c,d). These apparatuses permit environmental temperatures within the chamber up to 200 °C by electrical heaters (Figure 3b). To investigate the effect of temperature, several sandstone specimens were placed in the chamber at 75 or 125 °C for 1 h before the tests began, so that the notched crack propagated within rocks under higher temperatures. Other tests were performed at room temperature (~25 °C). The bedding planes of the shale specimens were set perpendicular (horizontal) or parallel (vertical) to the notched cracks to analyze the effect of the bedding orientation.

BD tests were conducted on at least three specimens in parallel with the same lithology, bedding orientation, and temperature, and the average strength was taken as the tensile strength for the corresponding set of conditions. The *K*_IC_ of each CCNBD specimen and the above average *σ*_t_ were used in Equation (3) to calculate the crack tolerance.

### 3.3. Experimental Results

**Figure 4:**
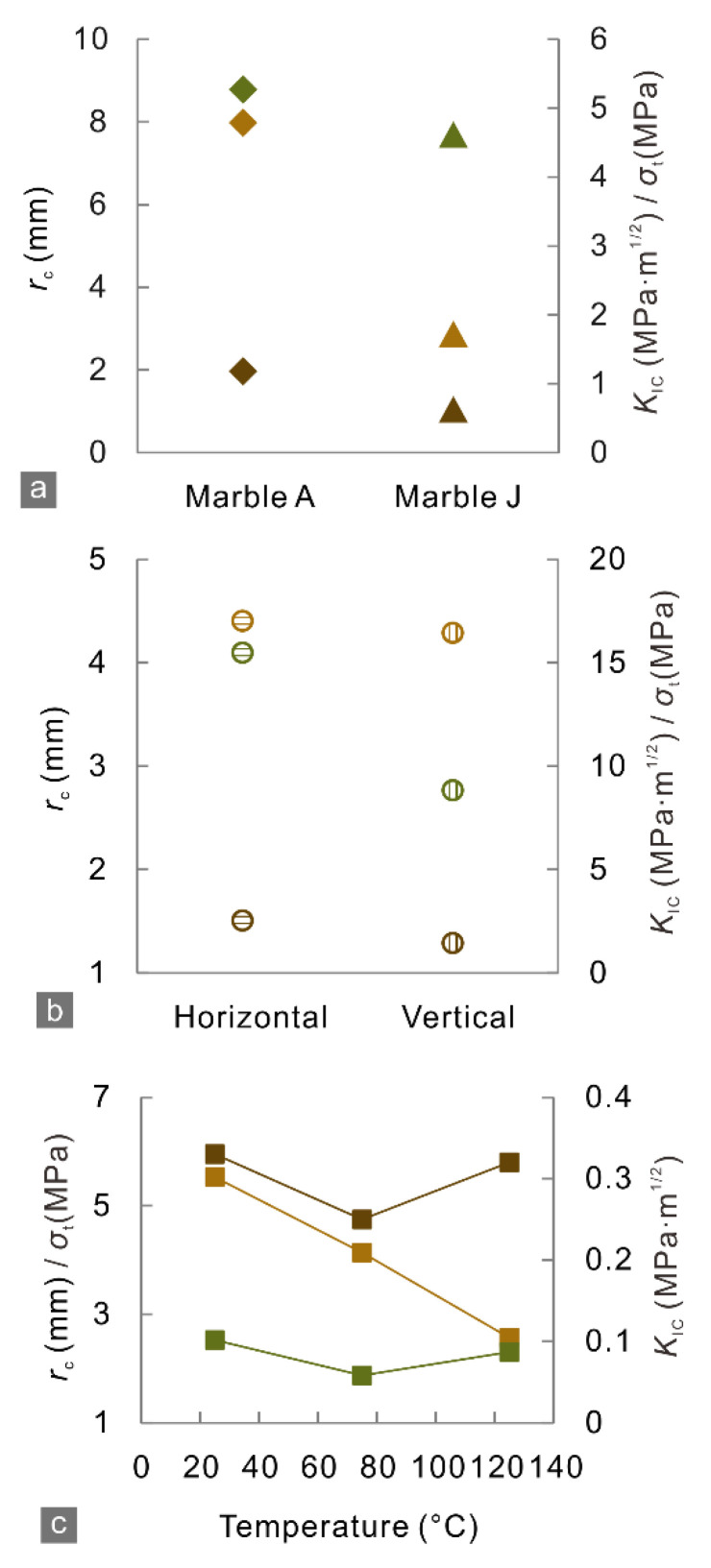
Mean tensile strength (brown), tensile fracture toughness (green), and crack tolerance (sienna) of the (**a**) marble A (diamond) and J (triangle), (**b**) shale (circle) with horizontal and vertical orientations and (**c**) sandstone (square).

**Figure 5:**
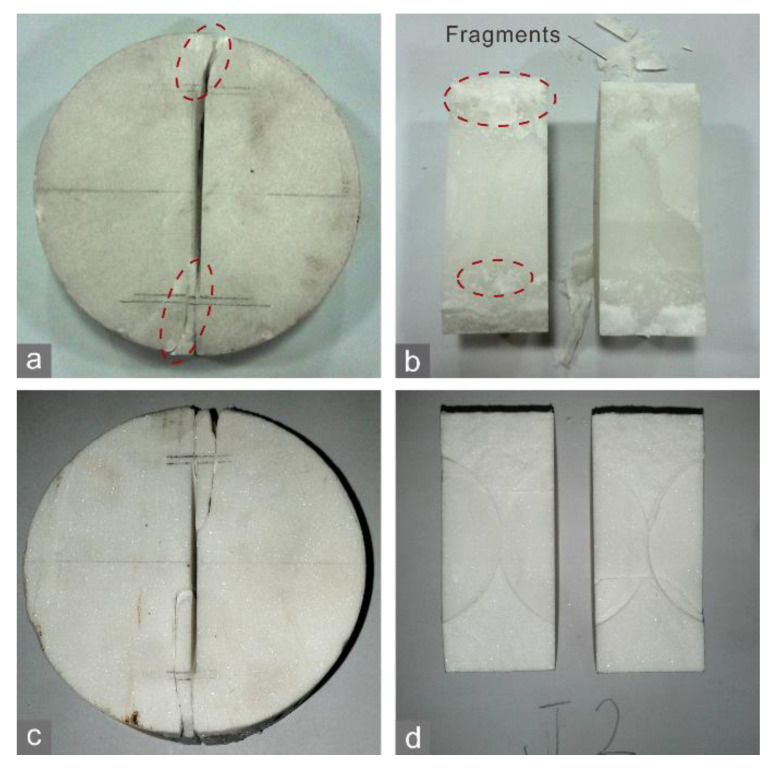
Crack morphologies of (**a**,**b**) marble A and (**c**,**d**) J. The red dashed ellipses in (**a**) denote white patches around the notched crack tips.

In the CCNBD test, the marble A specimens with stronger heterogeneity had greater tensile strength, fracture toughness, and crack tolerance than the marble J specimens, with relatively homogeneous small grains (Figure 4a). White patches indicating FPZs [25] appeared in front of the notched crack tips (Figure 5a) as the peak loads of the marble A specimens were approached. The patches corresponded to the sparkling areas on the rupture surface (Figure 5b), which may imply breaking cleavages of grains. However, such patches were not observed for the marble J specimens (Figure 5c,d), and neither were the discernible sparkling areas. Furthermore, the main cracks in the marble A specimens had branches causing more fragments (Figure 5b) while the crack in the marble J specimens propagated along a straight path (Figure 5c). These phenomena suggest that a more heterogeneous grain size corresponds to a larger crack tolerance and thus a stronger ability for crack generation.

**Figure 6:**
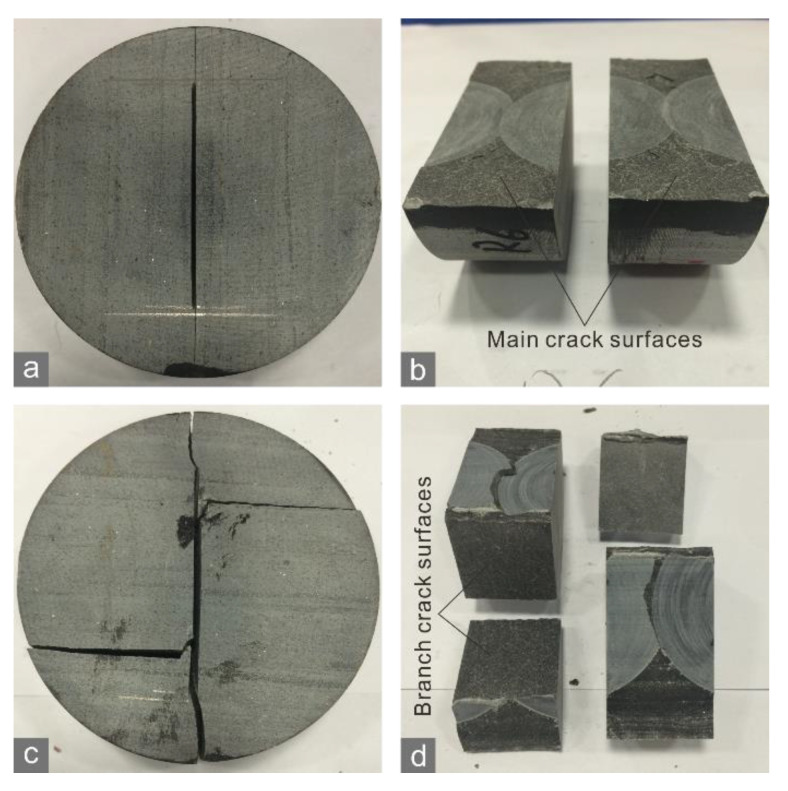
Crack morphologies of the shale CCNBD specimens with (**a**,**b**) horizontal and (**c**,**d**) vertical bedding orientations.

The mean crack tolerance of the shale specimens was less with a vertical bedding orientation than with a horizontal orientation (Figure 4b). The tensile strength and fracture toughness displayed similar variation trends with bedding orientation. Similar results can be acquired based on the data from Wang [26]. With a vertical orientation, the main crack of the specimen propagated along the bedding planes (Figure 6a), which generated a smooth rupture surface (Figure 6b). In contrast, with a horizontal orientation, the main crack spanned across bedding planes, and the path with steps was more irregular (Figure 6c,d). This is because the main crack was offset or even bifurcated when it encountered a bedding plane. The branch cracks were captured by bedding planes and then propagated along them, thereby their morphologies were smooth.

**Figure 7:**
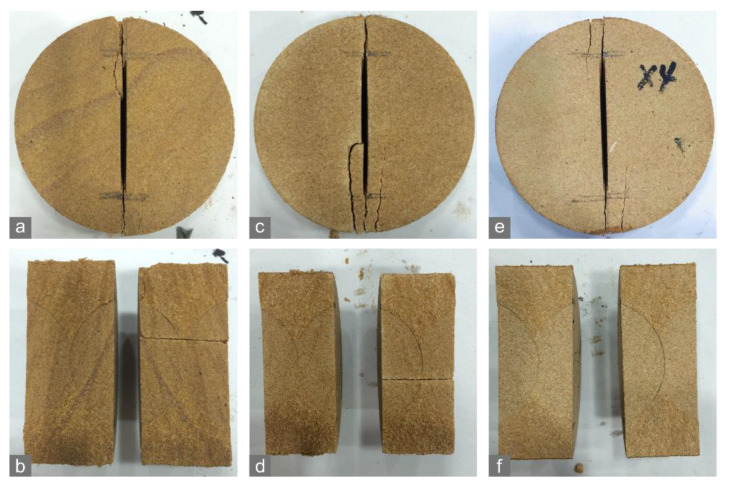
Crack morphologies of the sandstone specimens in the CCNBD tests at (**a**,**b**) 25 °C, (**c**,**d**) 75 °C, and (**e**,**f**) 125 °C.

The crack tolerance value of the sandstone specimens consistently declined as the environmental temperature rises from 25 °C to 125 °C, while the tensile strength and fracture toughness exhibited V-shaped trends within this temperature range (Figure 4c). It is difficult to identify changes in crack morphology with the rising temperature with the naked eye (Figure 7a,c,e). According to the edge of their rupture surface, we speculated that the main cracks in the specimens at 125 °C may propagate along less curved paths than the specimens at lower temperatures did (Figure 7b,d,f). The variations of the crack tolerance value and crack morphologies imply that high temperatures possibly reduce rock fracability.

The crack tolerance preliminarily showed an ability to address the aforementioned problems of previous fracability indices. Firstly, this index has a firm physical basis derived from the FPZ size, representing the nonlinearity of deformation due to micro-crack generation before the macroscopic propagation of the crack. Additionally, the formation of the FPZ is the inherent mechanical behavior of heterogeneous rock materials, and the FPZ size highly depends on the degree of heterogeneity as the previous [27] and present experiment results revealed. From the aspect of practicability, the crack tolerance value can be determined conveniently in the laboratory because BD and CCNBD tests are very common rock mechanical tests, and the small-size specimens they use can be easily obtained from cores. Finally, this index may characterize the effects of structure and loading conditions on fracability to an extent, as the tests on the shale and sandstone showed.

## 4. Numerical Analysis with the Discrete Element Method

### 4.1. Particle Flow Code

To test the rationality of the crack tolerance as an evaluation index for rock fracability, we adopt particle flow code in two dimensions (PFC^2D^), which is widely used for discrete element method (DEM). Rock was modeled as a dense packing of non-uniform-sized and inter-bonded circular particles using this method, and its mechanical behavior relied on the microscale properties and constitutive relations of the bonded contacts between the particles. Following Newton’s laws of motion, the force acting at each contact were updated with the particle movements during the simulation process, and the breakage of bonds representing crack generation [28] occurred when a component of the contact force satisfied a certain criterion.

**Figure 8:**
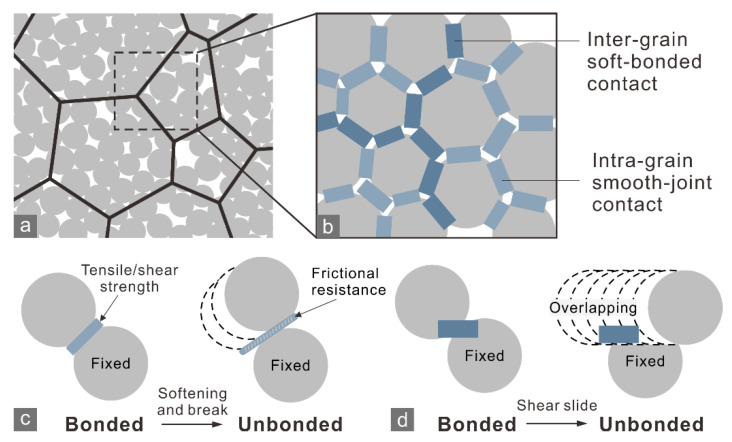
(**a**) Grain-based model for marble and (**b**) the implement of inter-grain soft-bonded and intra-grain smooth-joint contacts. Schematic of bonded and unbonded state for (**c**) soft-bonded and (**d**) smooth-joint contacts. (**b**) is the zoomed-in view of the dashed box in (**a**).

We used the experimental results for marble A and J as examples for the DEM simulation because the marble contained polygonal minerals >1 mm in size, which allowed us to implement a grain-based model (GBM) with a polygon-tessellation grain boundaries [29]. Such a model takes the mineral grain texture into account (Figure 8a), making a simulation more vivid. The modeling method for GBM of marble refers to [27]. Soft-bonded [30] and smooth-joint models [31] were employed to express bonded and unbonded behaviors characteristic of intra-grain and inter-grain contacts (Figure 8b–d), respectively.

### 4.2. Model Setup and Parameter Calibration

Based on the grain size distributions in Table 1, four circular 75 mm GBMs were created representing the marble A and J specimens in the BD and CCNBD tests. Each model comprised ~20,000 circular basic particles with a 0.2–0.3 mm radius. Since even a single mineral crystal is anisotropic along different atomic lattices [32], we set the strength and deformation parameters of the soft-bonded contacts to follow the Weibull distribution, and the shape parameter representing heterogeneity was set to 3 and 5 for marble A and J, respectively. A small value for the shape parameter indicates strong heterogeneity.

**Figure 9:**
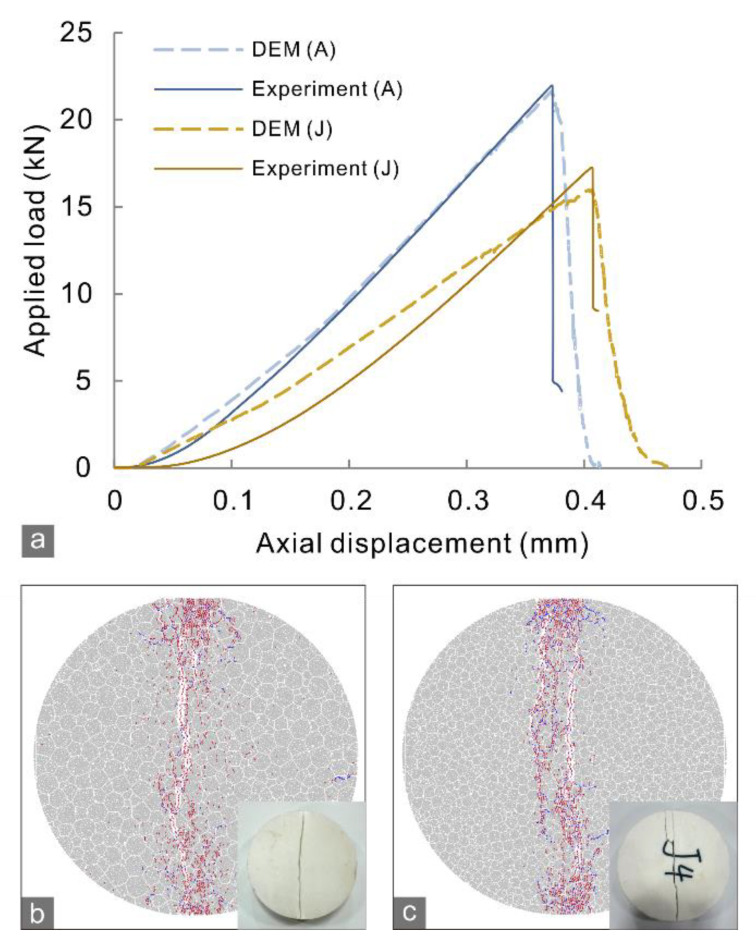
(**a**) Load–displacement relationship of experimental and numerical results for marble A and J in the BD test. (**b**,**c**) Crack morphologies of the marble A and J specimens in the numerical simulation of BD test and the corresponding experiment results.

Before conducting the simulation of the CCNBD tests, the microscale parameters of the particles and contacts required iteratively calibrating through trial and error referring to the BD test results and the previous work [27,33,34]. The GBMs of the BD specimens were positioned between two stiff walls representing the loading end and platform of a compression machine, and the walls moved toward each other at the same constant velocity to result in a quasi-static loading rate. The calibration completes until the simulated load–displacement curves and crack morphology fit well with the observations in the BD tests (Figure 9). The calibrated microscale parameters (Table 2) were used to simulate the marble specimens in the CCNBD test.

### 4.3. Simulation Results

**Figure 10:**
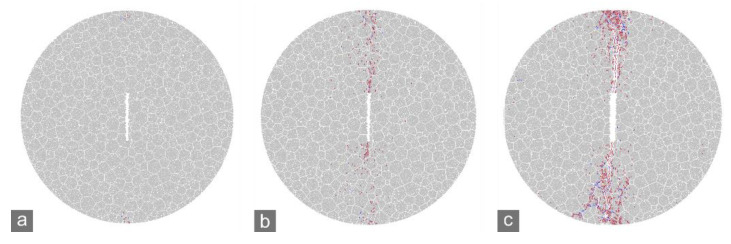
Simulated cracking process of marble A CCNBD specimen. (**a**) Cracking initiation at notched crack tips (~66% *P*_max_); (**b**) reaching *P*_max_; (**c**) specimen rupture.

**Figure 11:**
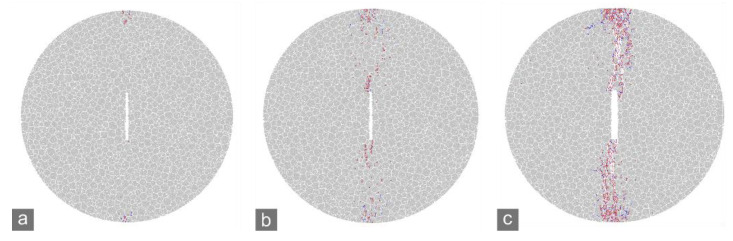
Simulated cracking process of marble J CCNBD specimen. (**a**) Cracking initiation at notched crack tips (~70% *P*_max_); (**b**) reaching *P*_max_; (**c**) specimen rupture.

In the numerical simulations of the CCNBD test, when the applied load reached a certain level, micro-cracking was initiated near the notched crack tips of the specimens (Figure 10a and Figure 11a). At the peak loads (*P*_max_) of the marble A and J specimens, the micro-cracks around the crack tips tended to coalesce to form new macroscopic cracks (Figure 10b and Figure 11b). After that, the notched crack propagated dramatically, which caused a rapid post-peak drop in the applied load and the specimen to rupture (Figure 10c and Figure 11c). Therefore, the preparation stage for dramatic propagation of a notched crack can be defined as from the initiation of micro-cracking to the reaching of the peak load, during which micro-cracks generate to develop the FPZ. The FPZ is the area near the crack tip with a dense micro-crack distribution when the peak load is reached that stays in the critical state of macroscopic rupture. As mentioned in Section 2, the crack tolerance is characterized by the size of the FPZ.

As the simulation results (Figure 10b and Figure 11b) showed, the micro-crack density at the vicinity of notched crack tip was especially high in the whole specimen, owing to the nonlinear deformation brought by stress concentration. The FPZ was assumed as a tip-centered circle with radius of *r*_c_ [14] that covered the area with high micro-crack density. Therefore, with increasing distance (radius) *R*_f_ from the tip and the diminishing intensity of the stress concentration, the deformation transitions from nonlinear inside the FPZ to quasilinear outside the FPZ, and thus the micro-crack density outside the FPZ declined to the background density of the rock [35].

**Figure 12:**
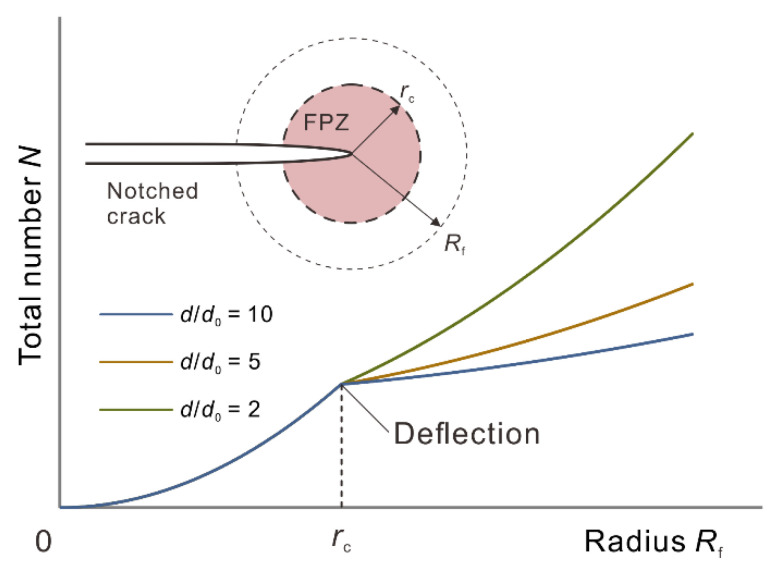
Variation trend of total micro-crack number inside tip-centered circles with various radii *R*_f_. The three curves correspond to cases under different ratios of *d*/*d*_0_.

To simplify the analysis, we assumed that the distributions of micro-crack inside and outside the FPZ are uniform but have different density. On the basis of this, the total micro-crack number *N* within a certain circular statistical range with *R*_f_ radius can be formulated as

(7) $$N\left(R\right)=\left\{\begin{array}{c} \pi ,\;\;|\;\;R_{f}<r_{c}\\ \pi r_{c}{}^{2}\left(d-d_{0}\right)+\pi R_{f}^{2}d_{0},\;\;|\;\;R_{f}\geq r_{c} \end{array}\right.$$

where *d* and *d*_0_ are the average micro-crack density inside and outside the FPZ, respectively. *N* displayed a positively correlation with *R*_f_; however, the curves of *N* deflected when *R*_f_ increased to *r*_c_ that defined the boundary of the FPZ (Figure 12). This is because the micro-crack density *d* inside the FPZ can be up to ~15 times as great as the background density *d*_0_ [36]; the increasing rate of *N* will decelerate once the statistical range extend outside the FPZ. Such a deflection became more identifiable with the increasing ratio of *d*/*d*_0_. Thus, the crack tolerance of the specimens can be determined by the radius corresponding to the deflection point.

**Figure 13:**
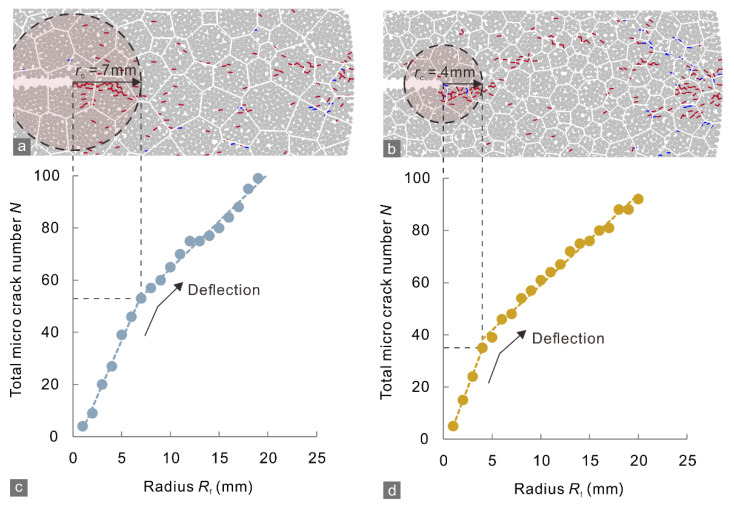
Micro-crack distributions near the notched crack tips in the (**a**) marble A and (**b**) J specimens at the peak applied load in the CCNBD simulation. (**c**,**d**) Total number of micro-cracks in tip-centered circles with different radii. The red and blue curves in (**a**,**b**) indicate intra- and inter-grain micro-cracks. The dashed lines in (**c**,**d**) are fitting lines for the distribution of the solid circles.

As the above calculation predicts, the micro-crack number *N* in Figure 10b and Figure 11b for the marble A and J specimens increased with *R*_f_, and the *N–R*_f_ curves deflected at radii of 7 and 4 mm, respectively (Figure 13), which were closed to the mean values of the crack tolerance of A (~8 mm) and J (~3 mm) measured by experiments. These results demonstrate that calculating the crack tolerance using the tensile fracture toughness and average tensile strength in Equation (3) leads to reliable results. The simulations also showed that marble A had a greater crack tolerance than marble J, and the FPZ of the former contained more micro-cracks than that of the latter preceding specimen rupture (Figure 13c,d). Correspondingly, the GBM of marble A generates 1473 micro-cracks in the whole loading process, more than that of marble J (1404). These results showed that a greater crack tolerance can represent a stronger ability to generate micro-cracks.

**Figure 14:**
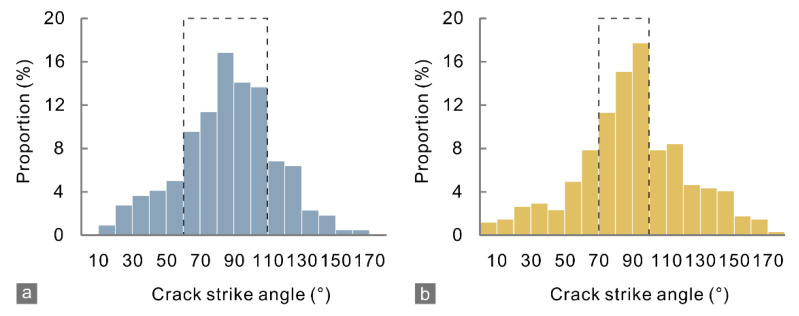
Strike angle distribution of micro-cracks in the CCNBD simulations for (**a**) marble A and (**b**) J, corresponding to Figure 10b and Figure 11b. The dashed boxes define the main distribution ranges.

The strike angle of micro-cracks (Figure 14) for the marble J mainly distributed in the range of 70–100°, which was narrower than that, 60–110°, for the marble A. This result suggests that more micro-cracks deviated from the loading direction (90°) in the marble A. The coalescence of such micro-cracks with various strike angles resulted in macroscopic cracks that propagated along irregular even branched paths and radiated from the notched crack tips (Figure 5 and Figure 10). Otherwise, the macro-cracks will develop primarily parallel to the loading direction, and thus their morphologies were less complex, as the marble J specimen showed. Therefore, these observations confirmed the assumption that fracability can be characterized by the crack tolerance.

## 5. Discussion

### 5.1. Effect of Grain Size on Crack Tolerance

The grain size greatly influences the cracking behavior of rocks [37]. Regarding rock consisting of grains with various size, the grain size greatly differs across parts of the rock specimen, so does the microscopic strength, which enhances the rock heterogeneity. This is why the heterogeneity index (shape parameter for the Weibull distribution) of marble A was set as smaller than that for marble J in the GBMs.

The microscopic strength in different parts of a strongly heterogeneous rock specimen can distribute in a wide range, so a small increment in the stress near a crack tip can easily cause micro-cracking within such rocks. Therefore, the initiation of micro-cracking was earlier, i.e., corresponding to a smaller ratio between the applied load and the peak load, in marble A than in marble J (Figure 10 and Figure 11). However, cracking can also be arrested easily because it probably encounters stronger local parts soon. Therefore, the rupture of strongly heterogeneous specimens will not occur until there are adequate micro-cracks to create FPZs and macroscopic cracks. In summary, strong heterogeneity strengthens the crack tolerance of rocks.

For rock specimens with a homogeneous grain size distribution, its microscopic strength in different parts can be generally closed to a certain level. Thus, only a few micro-cracks arise before the stress near the notched crack tips reaches that strength level. Once the strength is reached, the cracks propagate dramatically, which causes a rapid rupture. Macroscopic cracks spanning across the specimen form nearly instantaneously following the generation of a small FPZ. For these reasons, relatively homogeneous marble J had a smaller crack tolerance than marble A.

### 5.2. Effects of the Bedding Orientation and Environmental Temperature

With a vertical bedding orientation, micro-cracking naturally initiates within bedding planes in front of crack tips and propagates along them because the tensile strength of shale bedding planes is usually much smaller than that of layers between planes. Therefore, the tensile strength and fracture toughness were relatively low. Since the micro-cracks are limited to thin bedding planes, the corresponding crack tolerance is also small. With a horizontal bedding orientation, layers comprising various mineral grains make the distribution of the microscopic tensile strength near each tip more heterogeneous, so micro-cracking may be scattered among the layers. The behavior of micro-cracks can be complex when they cross bedding planes: cracks may branch along bedding planes and result in curved macroscopic cracks. Although hydraulic fracturing involves with many factors affecting the interaction between cracks and bedding planes [38], it is generally recognized that a crack propagating along a bedding plane is the most unfavorable situation for generating complex fracture networks [39,40].

Thermal treatment of sandstone leads to dehydration and the thermal expansion of minerals, which promotes the brittle–ductile transition of minerals [41]. Dehydration occurs at ~100 °C, which is when absorbed water escapes from the mineral surface [42], and enhances the friction and bonding strength between minerals. Thermal expansion takes effect when the temperature exceeds 100 °C and closes preexisting micro-cracks in rocks [43], which enhances the tensile strength of mineral grains and boundaries within a certain temperature range. Owing to these effects arising exceeding 100 °C, the sandstone at 125 °C had higher tensile strength and fracture toughness than that at 75 °C (Figure 4c). However, the dehydration and thermal expansion of minerals reduce the structural heterogeneity and rock fracability. In addition, fracability also weakens when sandstone transitions from brittle to ductile [44]. These observations suggest that increasing the temperature from 25 °C to 125 °C should be unfavorable for crack generation in sandstone. Such a negative effect of temperature was also observed in Longmaxi shale [45], a commercial shale gas reservoir in Chongqing, China. Considering that the downhole temperature, especially in deep and geothermal wells, can be much higher than the surface temperature, more attention should be given to the effect of temperature over a broader range on rock fracability.

### 5.3. Implications in Hydraulic Fracturing

The FPZ indicates nonlinear deformation (i.e., micro-crack generation) within a rock, which originates from rock heterogeneity. Nonlinear deformation diminishes and transitions into linear deformation with decreasing rock heterogeneity, which would reduce the FPZ size (i.e., crack tolerance). Complex cracks barely form in the absence of micro-cracks and their interactions. Because the formation of the FPZ is intrinsic to heterogeneous rock, the association between the FPZ size and heterogeneity is the physical basis for the crack tolerance as an evaluation index of fracability. The crack tolerance can reflect the effects of structural and environmental factors because they influence rock heterogeneity [46,47].

A large crack tolerance indicates many micro-cracks within the FPZ, which would tend to cause an irregular morphology of macroscopic cracks and wider zones of micro-cracks along both sides of cracks. These characteristics allow more cracks and pores in the rocks to connect with the main cracks during crack propagation driven by fluid. This increases the volume of cracks, which enhances the fluid conductivity of rock to exploit oil, gas, and geothermal resources.

The present study mainly provided laboratory observations on the effects of three factors to support the rationality of crack tolerance in reservoir fracability evaluation. However, the complexity of crack networks is dependent on various factors. Further investigations that consider other environmental and structural effects (e.g., magnitude and direction of crustal stress) are required to test this fracability index. On the other hand, the reliability and practical applicability of this index need to be further tested using rock specimens collected from reservoirs being exploited.

## 6. Conclusions

Aiming to the challenge of lacking a reliable index of fracability evaluation for hydraulic fracturing, we suggest that the crack tolerance, i.e., the maximum radius of the FPZ, may be used to characterize the fracability.

Crack tolerance originates from the unique heterogeneity of rock and inherent rules of crack generation, and thus it has a clear physical meaning and firm mechanical basis.

This index can be conveniently quantified in the laboratory using BD and CCNBD tests. We showed that the crack tolerance is positively correlated with the grain size heterogeneity and is negatively correlated with the environmental temperature (25–125 °C). The crack tolerance of shale specimens was greater with a horizontal bedding orientation than with a vertical orientation. Thus, crack tolerance can well characterize the effects of certain rock properties and environmental factors.

In summary, crack tolerance is promising in serving as a reliable evaluation index of rock fracability in terms of rationality and practicability, which has significant engineering implications for efficient hydraulic fracturing.

## Figures and Tables

**Table 1:** Microstructures of the rock specimens.

Microscopy Observation	Qualitative Description	Average Size (mm)	Grain Size Distribution
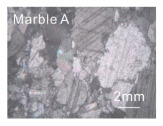	Medium-coarse grains	3.4	3–5 mm: 70% 1–3 mm: 30%
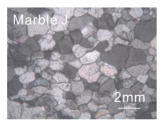	Medium grains	1.5	1–2 mm
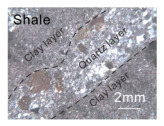	Fine grains	<0.1	/
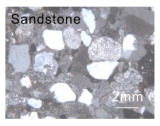	Coarse grains	2	1–3 mm

**Table 2:** Calibrated microscale parameters of marble A and J specimens.

Elements	Microscale Parameters	Marble A	Marble J
Particles	Density (kg/m^3^)	2690	2690
	Effective modulus (GPa)	70	70
	Normal to shear stiffness ratio	1.5	1.5
	Friction coefficient	0.6	0.6
Soft-bonded intra-grain contact	Effective modulus (GPa)	15.0	10.0
	Normal to shear stiffness ratio	2.0	2.0
	Friction coefficient	0.6	0.6
	Tensile strength (MPa)	28.0	18.0
	Cohesion (MPa)	112.0	72.0
	Friction angle (°)	45	45
	Softening factor	0.1	0.1
	Softening tensile strength factor	0.7	0.7
Smooth-joint inter-grain contact	Tensile strength coefficient	0.3	0.3
	Cohesion coefficient	0.8	0.8
	Friction angle coefficient	0.6	0.6
	Friction adjustment coefficient	0.5	0.5
	Normal stiffness coefficient	0.8	0.8
	Shear stiffness coefficient	0.15	0.15

## Data Availability

All the data required to evaluate the conclusions of this study are present in the paper. The authors will provide additional data related to this paper upon request.

## Nomenclature

List of symbols	
*a*_0_	Initial chevron notched crack length
*a*_1_	Final chevron notched crack length
ASTM	American Society of Testing Materials
*B*	Thickness of CCNBD specimen
*B*′	Thickness of BD specimen
BD	Brazilian disk
CCNBD	Cracked chevron notched Brazilian disk
*d*	Average micro-crack density inside FPZ
*D*	Diameter of CCNBD specimen
*D*′	Diameter of BD specimen
*d*_0_	Average micro-crack density outside FPZ
DEM	Discrete element method
FPZ	Fracture process zone
GBM	Grain-based model
ISRM	International Society for Rock Mechanics
*K*_I_	Tensile stress intensity factor
*K*_IC_	Tensile fracture toughness
*N*	Total micro-crack number
PFC	Particle flow code
*P*_max_	Peak applied axial load in CCNBD test
*P*′_max_	Peak applied axial load in BD test
*R*	Radius of CCNBD specimen
*r*	Polar radius
*r*_c_	Radius of circular FPZ
*R*_f_	Radius of circular statistical range
*R*_s_	Saw radius
*u*	Geometric constants
*v*	Geometric constants
XRD	X-ray diffraction
*Y* ^*^_min_	Critical dimensionless stress intensity
Greek symbols	
*α*_0_	Dimensionless parameter
*α*_1_	Dimensionless parameter
*α_B_*	Dimensionless parameter
*θ*	Polar angle
*σ*_1_	Maximum principal stress
*σ*_2_	Intermediate principal stress
*σ*_t_	Tensile strength
